# Transcription profiles of mitochondrial genes correlate with mitochondrial DNA haplotypes in a natural population of *Silene vulgaris*

**DOI:** 10.1186/1471-2229-10-11

**Published:** 2010-01-13

**Authors:** Hosam O Elansary, Karel Müller, Matthew S Olson, Helena Štorchová

**Affiliations:** 1 Institute of Experimental Botany, Academy of Sciences of the Czech Republic, Rozvojová 135, 165 00 Prague 6, Lysolaje, Czech Republic; 2 Department of Biology and Wildlife, University of Alaska at Fairbanks, Fairbanks, AK 99775, USA; 3 Institute of Arctic Biology, University of Alaska at Fairbanks, P.O. Box 757000, Fairbanks, AK 99775, USA

## Abstract

**Background:**

Although rapid changes in copy number and gene order are common within plant mitochondrial genomes, associated patterns of gene transcription are underinvestigated. Previous studies have shown that the gynodioecious plant species *Silene vulgaris *exhibits high mitochondrial diversity and occasional paternal inheritance of mitochondrial markers. Here we address whether variation in DNA molecular markers is correlated with variation in transcription of mitochondrial genes in *S. vulgaris *collected from natural populations.

**Results:**

We analyzed RFLP variation in two mitochondrial genes, *cox1 *and *atp1*, in offspring of ten plants from a natural population of *S. vulgaris *in Central Europe. We also investigated transcription profiles of the *atp1 *and *cox1 *genes. Most DNA haplotypes and transcription profiles were maternally inherited; for these, transcription profiles were associated with specific mitochondrial DNA haplotypes. One individual exhibited a pattern consistent with paternal inheritance of mitochondrial DNA; this individual exhibited a transcription profile suggestive of paternal but inconsistent with maternal inheritance. We found no associations between gender and transcript profiles.

**Conclusions:**

Specific transcription profiles of mitochondrial genes were associated with specific mitochondrial DNA haplotypes in a natural population of a gynodioecious species *S. vulgaris*.

Our findings suggest the potential for a causal association between rearrangements in the plant mt genome and transcription product variation.

## Background

Despite numerous investigations of the structure and dynamics of the plant mitochondrial (mt) genome over the last decades, little is known about whether patterns of DNA sequence variation are associated with variation in transcription products in plants from natural populations. Unlike animals that possess a small, compact, gene-dense, circular mt genome, land plants have mt genome which is organized as a collection of circular and linear molecules of various sizes [1]. Plant mt genomes are known to rapidly change copy number and gene order, which results in insertions and deletions in both functional and non functional regions. The occasional coexistence of at least two different copies of mt DNA in the same individual, which is termed heteroplasmy, creates the opportunity for recombination or re-association among different mt lineages [2].

The transcription of plant mt genes is complex and well characterized in only a few model systems. Transcription is performed by a phage-type RNA polymerase encoded by the nucleus [3] and the presence of multiple promoters is a common feature of plant mt genes [4,5]. Splicing, editing and processing of transcript termini are all involved in maturation of mRNA in plant mitochondria [6,7]. Moreover, self-splicing group II introns are present in at least ten plant mt protein coding genes. Variation in the sizes of transcripts from the same mt gene has been found in several studies [8,9]. Mt RNA profiles have been shown to depend on the developmental stage of the plant [10] and transcription profiles have been found to be species or lineage specific in wheat, rye, triticale, and *Arabidopsis thaliana *[7,11,12]. Additionally, differences in mt gene transcription and translation between females and hermaphrodites have been used to discover candidate genes for cytoplasmic male sterility (CMS) [9,13]. To date, however, there has been very little investigation of naturally occurring variation in transcript sizes and whether this variation is related to mt genome arrangements for any species.

*Silene vulgaris*, a Eurasian short-lived perennial, exhibits a wide range of sex ratios in natural populations and has become a model for studies of the population genetic consequences of gynodioecy [14,15]. Diversity in the mt genome in natural populations is better characterized for *S. vulgaris *than in any other plant species [16-18]. Mt DNA markers (RFLP of *coxI *region) have been applied, for example, to demonstrate a high polymorphism of *S. vulgaris *in the USA [16] and Central Europe [17]. The high level of RFLP polymorphism is accompanied by very high substitution rates in coding regions of some mt genes [19]. High nucleotide diversity is also associated with the gynodioecious reproduction system in the genus *Silene *[20], although no CMS related gene has been discovered in *Silene*.

We hypothesize that there may be causal links between mitochondrial rearrangements and variation in transcription profiles. Regulatory motifs are often located in the gene flanking regions, which are frequently the sites of intra- or inter-genomic rearrangements [8,20]. These rearrangements could lead to the changes in gene transcription patterns. For this reason, we investigated whether polymorphism in mt DNA is associated with polymorphism in gene transcription profiles. We know of no study to date that has documented variation in transcription profiles of plant mt genes in natural populations. Although the mt genome is primarily maternally inherited [21], rare paternal inheritance of the mt genome has been described in natural populations of *S. vulgaris *[22,23]. This phenomenon offers opportunity to correlate transcription profiles and genetic background.

In the present study we investigate whether transcript profiles of two mt genes correlate with mt DNA haplotypes. The *cox1 *and *atp1 *genes were chosen based on the previous reports of their high polymorphism in *S. vulgaris *[16,17]. We describe mt DNA variation and transcription profiles of the *cox1 *and *atp1 *genes in offspring of maternal plants collected from one highly diverse population of *S. vulgaris *in Central Europe. We also compare transcript patterns of the two genes between the genders with the aim of identification of possible candidates for CMS genes in *S. vulgaris*.

## Results

### Variation in DNA patterns among families

**Figure 1:**
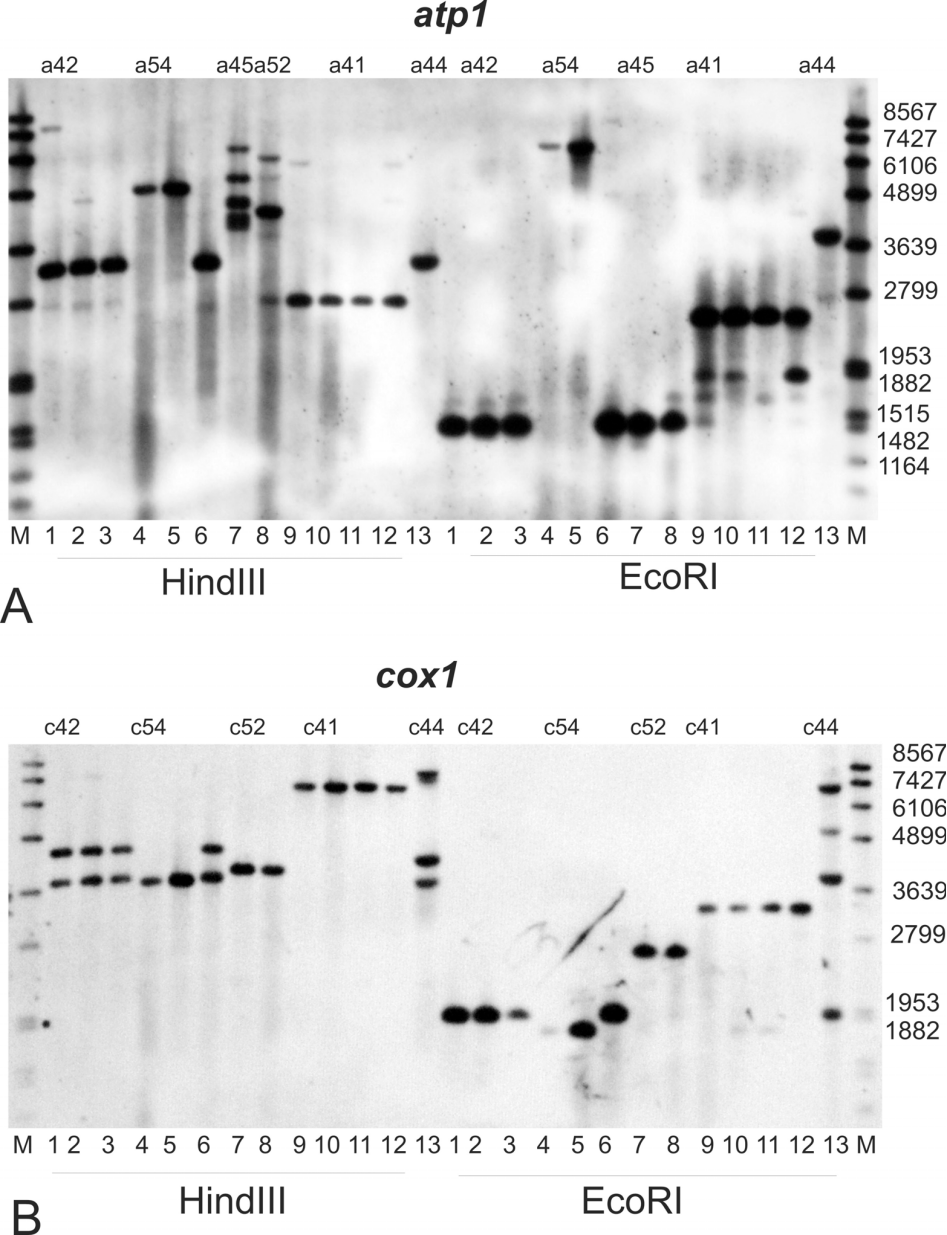
**High variation in mitochondrial Southern-RFLP patterns among representatives of ten families from the *S. vulgaris *population Kovary Meadows**. Total DNA was digested with HindIII and EcoRI and hybridized with DIG labeled *atp1 *(**A**) and *cox1 *(**B**) probes. The individual plants belong to the following families: 1. Kov42, 2. Kov53, 3. Kov43, 4. Kov53 (paternal transmission), 5. Kov54, 6. Kov53, 7. Kov45, 8. Kov52, 9. Kov52 (paternal transmission), 10. Kov41, 11. Kov46, 12. Kov51, 13. Kov44. S - DNA molecular size marker. Southern-RFLP haplotypes are assigned above the panels.

Two methods were adopted to analyze mt DNA variation in *S. vulgaris*: 1) Southern-RFLP's, which screened RFLP variation in regions flanking the *atp1 *and *cox1 *genes, and 2) PCR-RFLPs that screened RFLP variation in the coding regions of the same genes. Among 331 offspring distributed across 10 families (18-39 plants per family) of *S. vulgaris *originating from Kovary meadows near Prague (Table 1), Southern hybridization revealed 5 different RFLP haplotypes in the *cox1 *flanking regions (designated c41, c42, c44, c52 and c54; figure 1b, Table 1) and 6 different haplotypes in the *atp1 *flanking regions (a41, a42, a44, a52, a45 and a54; figure 1a, Table 1). Additional faint bands were observed in nearly all RFLP patterns (94%) in at least one combination probe/restriction enzyme. The *cox1 *Southern-RFLP haplotype c42 matched the haplotype L described from a previous study in the same population [17], but all other Southern-RFLPs differed from those previously described [17].

**Table 1:** PCR-RFLP and Southern-RFLP mt DNA haplotypes found among ten maternal plants of *S. vulgaris *growing in Kovary Meadows (Czech Republic).

*PCR - RFLP*		*Southern - RFLP* *Numbers indicate approximate fragment lengths (kb) of major bands*			
***cox1***	***atp1***	***cox1*** **EcoRI**	***cox1*** **HindIII**	***atp1*** **EcoRI**	***atp1*** **HindIII**
Kov**B'**	Kov**C**	**c41 **3.2	**c41 **7.3	**a41 **2.5;1.8	**a41 **2.8
Kov**B'**	Kov**C**	**c54 **1.7	**c54 **3.8	**a54 **7.2	**a54 **5.1
Kov**A'**	Kov**B**	**c42 **1.9	**c42 **4.6; 3.9	**a42 **1.5	**a42 **3.1
Kov**A'**	Kov**B**	**c44 **7.3; 5.0; 3.8;1.9	**c44 **8.3; 8.0; 4.1;3.8	**a44 **3.8	**a44 **3.2
Kov**A'**	Kov**A**	**c52 **2.6	**c52 **3.9	**a45 **1.5	**a45 **6.4; 5.5; 4.6; 3.8
Kov**A'**	Kov**A**	**c52**	**c52**	**a45**	**a52 **6.1; 4.4

Sequencing of coding regions revealed 2 *cox1 *variants (designated KovA' and KovB') and 3 *atp1 *variants (KovA, KovB, KovC) (Table 2), which were also distinguishable by restriction digestion of PCR fragments. Twenty one nucleotide sequence differences were identified within the 1218 bp alignment of the 3 *atp1 *haplotypes. Haplotype KovA, present in the Kov45 and Kov52 families, differed from the haplotypes KovB and KovC by 18 and 16 differences, respectively. Three of these nucleotide sequence differences were non-synonymous. In contrast, the *cox1 *sequences were very similar - haplotypes KovA' and KovB' differed by two synonymous nucleotide differences within 1400 bp. All *atp1 *and *cox1 *sequences matched at least one record previously deposited in GenBank. For instance, the sequence of the *atp1 *haplotype KovB was identical with the *atp1 *haplotype A (DQ422872) described in [22].

**Table 2:** Family codes and mt DNA haplotypes.

*Family*	*PCR - RFLP* *haplotype*		*Southern - RFLP* *haplotype*				*Number of individuals*
		
	*cox1*	*atp1*	*cox1* EcoRI	*cox1* HindIII	*atp1* EcoRI	*atp1* HindIII	
Kov 41	Kov**B**	Kov**C**	**c41**	**c41**	**a41**	**a41**	18
Kov 46	Kov**B**	Kov**C**	**c41**	**c41**	**a41**	**a41**	32
Kov 51	Kov**B**	Kov**C**	**c41**	**c41**	**a41**	**a41**	26
Kov 54	Kov**B**	Kov**C**	**c54**	**c54**	**a54**	**a54**	38
Kov 42	Kov**A**	Kov**B**	**c42**	**c42**	**a42**	**a42**	32
Kov 43	Kov**A**	Kov**B**	**c42**	**c42**	**a42**	**a42**	38
Kov 53 *	Kov**A**	Kov**B**	**c42**	**c42**	**a42**	**a42**	38
Kov 44	Kov**A**	Kov**B**	**c44**	**c44**	**a44**	**a44**	36
Kov 45 *	Kov**A**	Kov**A**	**c52**	**c52**	**a45**	**a45**	39
Kov 52 *	Kov**A**	Kov**A**	**c52**	**c52**	**a45**	**a52**	34

The families originated from ten maternal plants growing in Kovary Meadows (Czech Republic). Families comprising at least one individual differing from mother or siblings in mt DNA markers are marked (*).

### Non-maternal transmission of organellar markers

Three individuals were identified that differed from their siblings and maternal parents in mt DNA haplotypes. For instance, individuals in lanes 2, 4, and 6 in figure 1 are siblings from the Kov53 mother. Individuals 2 and 6 carried the same mt type as their mother, but the individual in lane 4 (Kov53-3) carried different mt haplotype. Another example was individuals in lanes 8 and 9 in figure 1. Although these siblings were grown from seeds collected from the Kov52 mother, the mt haplotype of the individual in lane 9 (Kov52-23) did not match that of the mother. Finally, one plant in the Kov45 family (data not shown) was identical to its siblings except for a unique *cox1 *Southern RFLP pattern that did not correspond to any other haplotype in this study. Because the mother plants shared mt markers with the majority of offspring and the Southern-RFLP band intensity was strong in all cases, we interpret these patterns as either evidence of paternal transmission from unknown pollen donor in natural population, or a sudden increase in genome copy number by means of lineage sorting or substoichiometric shifting.

The presence of a unique SmaI restriction site allowed us to apply the knock-back approach introduced by [22] to verify whether the unique *atp1 *haplotype in the progeny from the mother Kov52 was present in a low frequency in the mother as well. The amplification of maternal DNA, pre-digested with SmaI, generated no bands; a result that can be interpreted as an absence of the progeny haplotype in the Kov52 mother and favors the paternal transmission hypothesis for this case. Suitable restriction sites in *atp1 *genes were absent in remaining two cases of different haplotypes in families, preventing application of "knock-back" approach. Based on these results we estimate the rate of paternal transmission in Kovary Meadows to be between 0.3% (assuming only 1 paternal transmissions/331 plants screened) and 0.9% (assuming all three mismatch progeny resulted from paternal transmission).

### Heteroplasmy in the Kov52 and Kov45 families

We also applied the knock-back approach to address the presence of heteroplasmy in all of the progeny from the two families that carried *atp1 *haplotype KovA (Kov52 and Kov45). Seven individuals (20%) in the Kov52 family carried low copy numbers of the *atp1 *allele without the SmaI site that is present in haplotype KovA. Because the mother plant Kov52 was homoplasmic for KovA, these copies were most likely paternally transmitted. Eighteen plants (46%) from the Kov45 family also contained an additional *atp1 *variant in low copy number. Because the mother plant 45 also was heteroplasmic, we can estimate that 54% of siblings in Kov45 family lost the rare non-KovA *atp1 *allele through genetic drift and mitochondrial sorting within or across generations.

The two families with the *atp1 *haplotype KovA (Kov52 and Kov45) also showed variation in Southern RFLP of *atp1 *among progeny. While the major bands of the RFLP patterns were uniform, additional bands of the same or fainter intensity appeared in some progeny, but not in others. Four individuals with an extra band as intense as the major bands were found among 39 siblings in the Kov45 family and 20 individuals with an extra band slightly fainter than the major bands were found among 34 siblings in the Kov52 family. Variation in Southern-RFLP band intensities was also observed in all the families. For instance, the strength of the 1.8 kb EcoRI fragment corresponding to the *atp1 *gene varied in Kov41, Kov46 and Kov51 families (figure 1). Another example is HindIII and EcoRI *cox1 *Southern RFLP of the members of the Kov44 family, with varying intensities of the second band (figure 1).

### Within-individual DNA variation

**Figure 2:**
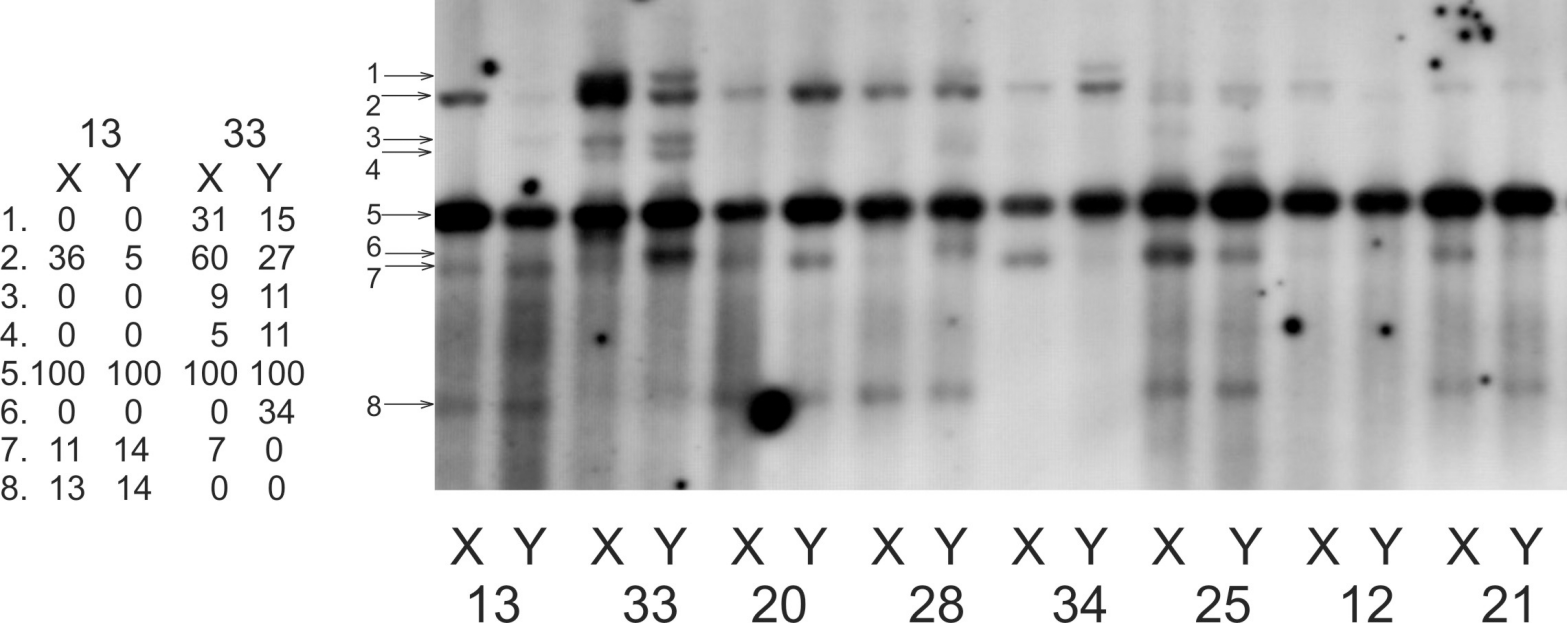
**Within-individual variation in the mt Southern-RFLP patterns in the family Kov52**. DNA was digested with HindIII and hybridized with DIG labeled *atp1 *probe. X and Y denote two different branches from the same individual identified by the number. Arrowheads point to the bands which intensities were quantified. The values of band intensities related to the strongest band (number 5) and expressed in % are shown at left.

Southern-RFLP patterns from leaf tissues collected from two different stems on the same plant were analyzed from 34 plants in the Kov52 sibship (figure 2). Within-individual differences in plants13 and 33 are clearly visible in figure 2. For example, the intensity of band 2 differs seven-fold between two branches of plant 13 and there was a two-fold difference in band intensity for band 1 on different stems from plant 33. In all, we detected within-individual band intensity variation in 5 of 34 plants screened from the Kov52 family. These results indicate the strong potential for sorting of different mitochondrial genomes into different branches during plant development to cause changes in the copy number mt genomes in different stems of the same plant. The branches differing in Southern-RFLP profiles produced flowers of the same gender.

### Transcription profiles of mt genes

**Figure 3:**
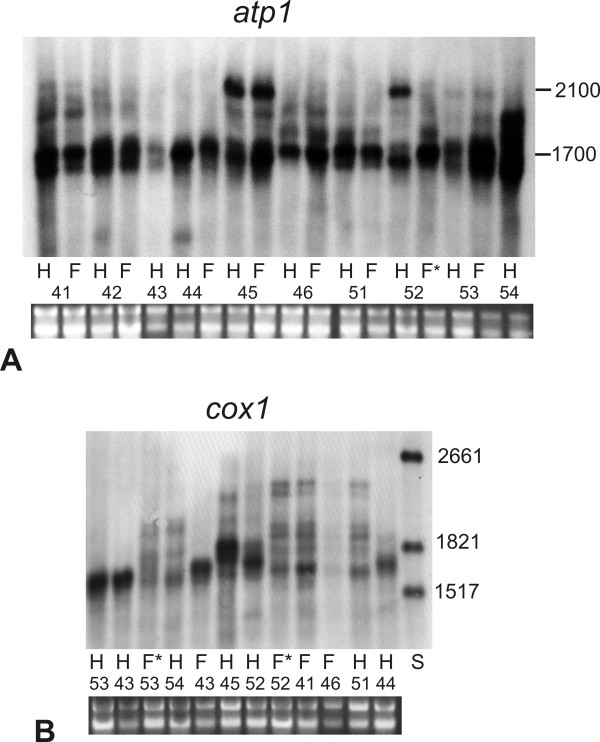
**Variation in mt transcription profiles among representatives of ten families from the *S. vulgaris *population Kovary Meadows**. Total RNA was transferred to the membrane and hybridized with DIG labeled *atp1 *probe (**A**) or *cox1 *probe (**B**). H - hermaphrodite; F - female; F* - individuals possessing mt DNA haplotype different from siblings show also different transcription patterns which correspond to the specific mt haplotypes. Family codes are written below each Northern. Ribosomal RNAs corresponding to the specific RNA samples are visualized below the panels. The numbers on the side indicate RNA molecular size (nt).

**Figure 4:**
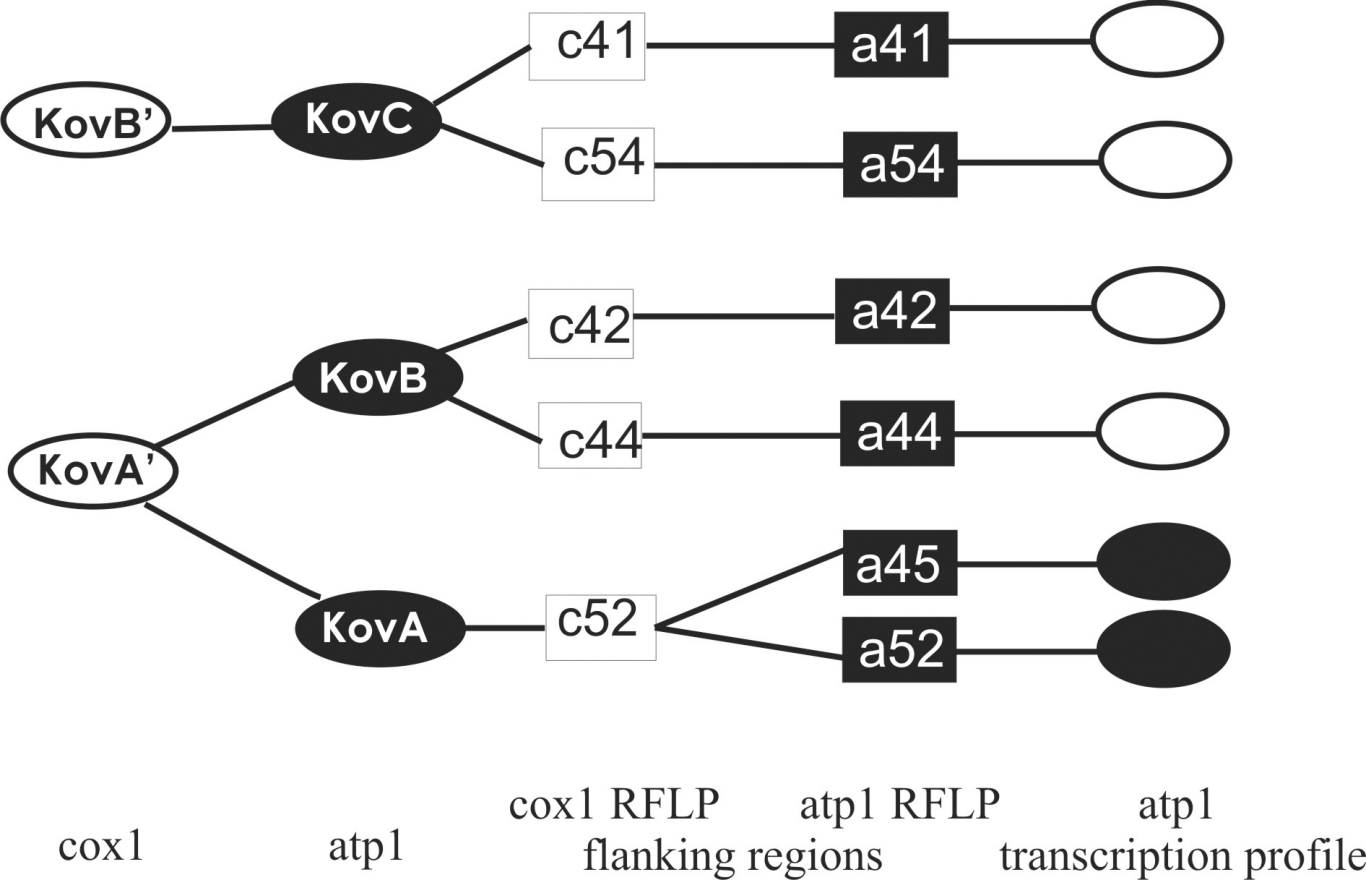
**Relationships among mt DNA haplotypes derived from coding and flanking regions of the *cox1 *and *atp1 *genes and transcription profiles of these genes as revealed in *S. vulgaris *population from Kovary Meadows**. Black circles denote transcription profiles possessing a strong additional band of the size about 2.1 kb. The *cox1 *PCR-RFLP haplotypes are designated KovA' and KovB', the *atp1 *PCR-RFLP haplotypes are designated KovA, KovB, KovC. The *cox1 *Southern RFLP variants are designated c41, c42, c44, c52, and c54. The *atp1 *Southern RFLP variants are designated a41, a42, a44, a52, a45, and a54.

To investigate variation in mt transcript patterns in *S. vulgaris *we performed Northern hybridizations. Figure 3a demonstrates differences in transcription profiles of *atp1 *across the families. All individuals showed a strong band at 1.6 kb. In addition, plants from the Kov45 and 52 families (sharing the *atp1 *KovA haplotype - figure 4) displayed an additional strong band about 2.1 kb. Additional weak bands occurred in all the individuals except for members of the Kov44 family, all of which possessed just one visible 1.6 kb band.

The additional bands showed variable intensities among the members of the same family, and their presence or absence was sometimes hard to confirm. Therefore, we chose the presence/absence of 2.1 kb extra band to define the *atp1 *transcription profile, as this character was highly reproducible. The *cox1 *profile (figure 3b) also showed variation in transcription patterns among individuals (e.g. six bands in the families Kov41, 46 and 51, which shared the same Southern-RFLP haplotype c41).

**Figure 5:**
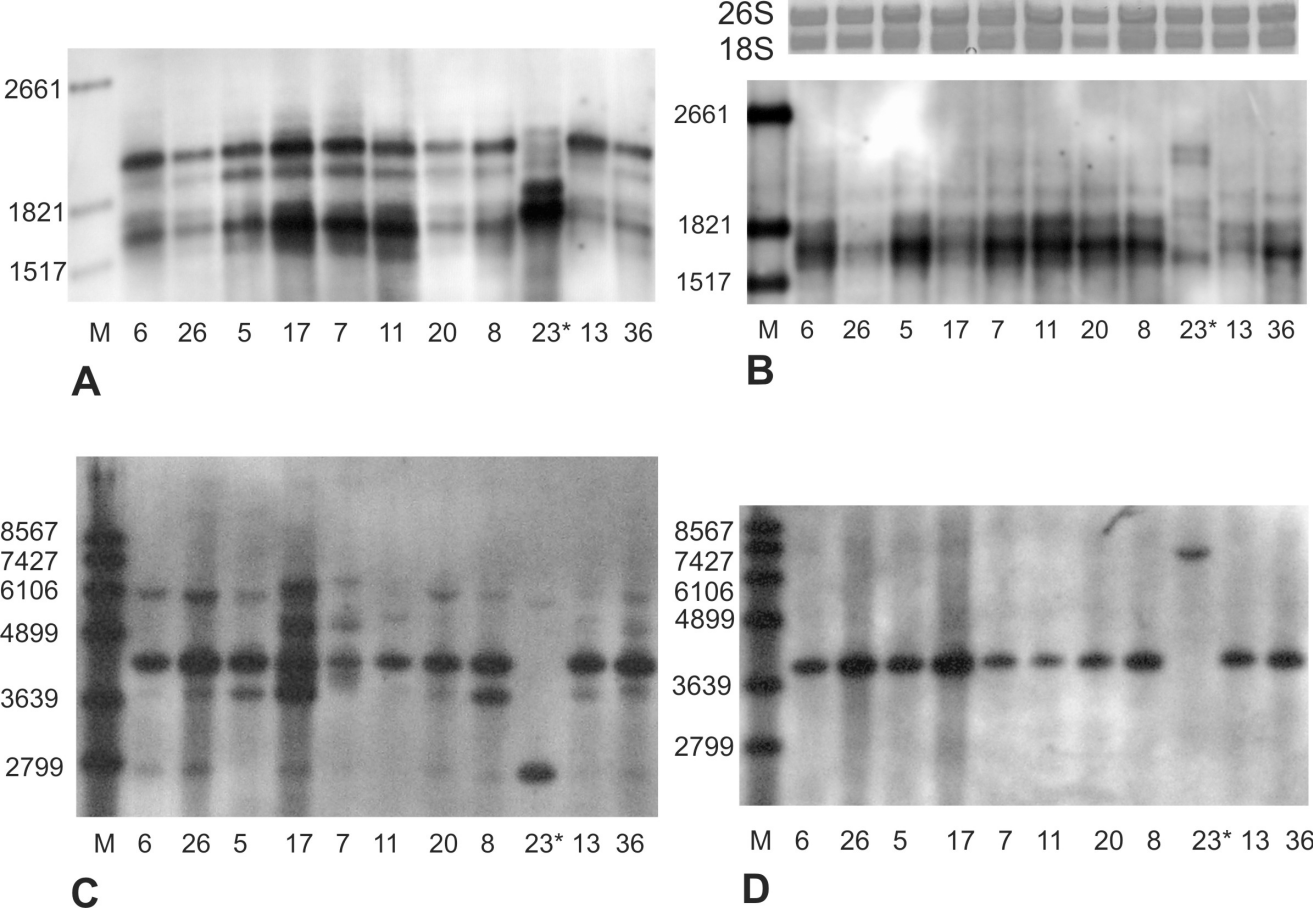
**The comparison of mt transcription profiles and Southern-RFLP patterns in a Kov52 sibship**. Total RNA was transferred to the membrane and hybridized with DIG labeled *atp1 *(**A**) or *cox1 *(**B**) probes. Ribosomal RNA stained by Ethidium bromide and corresponding to particular RNA samples is shown above *cox1 *transcript profile. DNA was digested with HindIII and hybridized with DIG labeled *atp1*(**C**) or *cox1 *(**D**) probes. The numbers denote individual plants. The plant 23* shows both different RFLP pattern and transcription profile from its siblings. S - DNA or RNA molecular size marker.

The transcription of mt genes starts from multiple promoters and primary transcripts undergo a complex maturation process [3-7]; therefore, we wondered whether the 2.1 kb *atp1 *band was associated with a specific DNA haplotype or arose as a result of the different developmental changes leading to floral buds in different individuals. We extracted total RNA from the floral buds of various sizes (1 to 4 mm) from 22 individuals from the Kov52 family and 23 plants from the Kov45 family. As shown in figure 5a, all but one plant shared the 2.1 kb band. Interestingly, the plant without the 2.1 kb band also differed from its siblings in mt DNA Southern-RFLP patterns, indicating, perhaps, co-paternal inheritance of both the mt DNA and transcriptional machinery (figure 5c). Similar patterns of transcription profiles were observed after rehybridization of the same membrane with a *cox1 *probe (figure 5b). Different *atp1 *and *coxI *transcription profiles were reproducibly associated with the *atp1 *PCR-RFLP DNA haplotype KovA, occurring in the Kov45 and Kov52 families (figure 4). The among-individual variation in minor Southern-RFLP bands (figure 5c) were not correlated with specific *atp1 *transcription profile. Another individual differing in both mt DNA haplotype and transcription profile from its siblings was found in the family Kov53. This plant shared DNA haplotype and transcription pattern with Kov54 (figure 3b).

We compared *atp1 *transcription profiles between 12 females and 12 hermaphroditic plants from Kov45 and Kov52 families, which inherited mt genomes from their mothers. An extra *atp1 *2.1 kb band was present in all of them, but its intensity in relation to the 1.6 kb band varied (figure 5). The relative strength of the *atp1 *2.1 kb transcript did not correlate with gender. Thus, the presence of the *atp1 *2.1 kb band was associated with the mt DNA haplotype and not gender, whereas its abundance was influenced by unknown factor(s).

## Discussion

### Trancription profiles correlate with mt DNA haplotypes

We have shown that variation in transcription profiles for *atp1 *and *cox1 *were correlated with mt DNA sequence variation in individuals from a single population of *S. vulgaris *in central Czech Republic. These associations are strong. The only individual that showed transcription profiles different from its siblings among 55 members of Kov45 and Kov52 families (figure 5) also differed from its siblings in all mt DNA markers analyzed. A similar association between mt DNA haplotype and transcript profile was also found in a progeny in the Kov53 family which shared both haplotype and transcript pattern with Kov54 family. This within-family variation in transcription profiles and mt DNA sequence likely resulted from rare paternal inheritance of the mitochondrial genome in these individuals and suggests that factors in the mitochondrial genome have regulatory influences on the mitochondrial genes than can be co-inherited. We caution, however, that because the maternal plants were field pollinated and we do not know pollen donors, we cannot completely exclude the possibility that the individuals with paternally transmitted mt genome also inherited nuclear gene(s) responsible for different transcription patterns.

Little is known about the mechanisms generating variation in mitochondrial gene transcription profiles. One potential mechanism for generating this variation is the rearrangement of the mt genome to move coding regions to a position proximate to a different promoter, resulting in novel mRNA types. For example, additional start sites, introduced by DNA rearrangements, were responsible for altered transcript sizes of six mt genes in *Beta vulgaris *[8]. Additionally, variation in 5' mRNA ends was responsible for transcript polymorphism in *A. thaliana *[6,7]. Finally, co-transcription of *nad6 *with an unknown ORF led to the generation of a large transcript occurring in a CMS lineage of *Mimulus guttatus *[9].

The transcription pattern of mt genes may also vary with the developmental stage [10], however, our observations did not support this process because RNA extracted from the flower buds of various ages from the individuals of the same haplotype shared a similar transcription profile. We recognize that our study does not conclusively prove that transcriptional patterns of mt DNA genes cannot change through the course of development as a result of mitochondrial rearrangements. If mitochondrial rearrangements during development are quite rare, it may require a serendipitous circumstance to detect the correlated changes in mt gene rearrangements and transcriptional patterns.

We also found no association between gender and the transcription pattern. The same number of bands was found in the *cox1 *and *atp1 *transcript profiles of females and hermaphrodites in all families and the relative intensities of particular bands did not differ in a consistent manner between females and hermaphrodites. Thus we did not find differential transcription between the two genders which would relate particular transcripts to CMS [8,9,13]. At the same time we cannot exclude, that some of the *cox1 *and *atp1 *genes are involved in CMS and their expression is regulated in gender-specific manner as described in [24].

### Paternal transmission of mt genome

Because the plants grown from the field pollinated seed had unknown fathers, we could not provide direct evidence for paternal transmission of mt markers by comparing the mt genotypes between progeny and the father. Instead, we assume that paternal inheritance was responsible for the mt haplotype of the particular plant if (1) the mt DNA markers of this individual differed from its siblings, if (2) its mt PCR-RFLP haplotype was not present in maternal plant even in trace amount, and if (3) it differed in all mt markers analyzed, which made paternal inheritance more parsimonious explanation than substoichiometric shifting [25,26].

We observed two individuals (Kov52-23, Kov53-3) that differed from their siblings in all mt markers; another plant (Kov45-19) differed in *cox1 *Southern-RFLP only. Because "knock-back" methods [22] found no heteroplasmy in the Kov52 mother, the different mt DNA haplotypes in Kov52 family could not have been inherited from the mother. Therefore, paternal transmission was the simplest explanation. The lack of suitable restriction sites prevented similar tests in the remaining two plants. We could therefore apply all the above mentioned criteria to the one individual only (Kov52-23), while the paternal inheritance in remaining two plants could not be either confirmed or excluded. The occurrence of 1-3 individuals (about 0.3-1.0% cases of paternal transmission) among 331 offspring is comparable to 4% of non-maternal offspring revealed among 318 *S. vulgaris *plants by [22]. Rare paternal inheritance (1.9%) of mt markers in a natural population of *S. vulgaris *has been recently documented by [23].

### Heteroplasmy does not influence transcription profiles

We interpret the results of our PCR-RFLP knock-back experiments and the presence of multiple bands in Southern banding patterns as evidence of mitochondrial heteroplasmy within individuals. Multiple bands in the Southern-RFLP profiles might also have been derived from additional gene copies, partial non-functional duplications, or from chimeric genes [2]. Besides major bands, faint to moderately strong bands were present in nearly all Southern-RFLP patterns; we interpret these weaker bands as low-copy molecules of mt DNA [25,26]. Moreover, differences in Southern-RFLP banding patterns between the branches of the same plant were dramatic. We suggest that such variation in frequencies of different markers arose from random sorting of mt genomes during vegetative growth of heteroplasmic individual [27]. However, rearrangements of mt DNA by a process similar to substoichiometric shifting [25,28] during development cannot be completely excluded. We found no variation in transcript profiles that were associated with heteroplasmy. However, our Northern hybridization experiments detected mt transcripts in total RNA, without distinguishing the relative contribution of specific mt genomes of the same individual. The differentiation and quantification of the transcripts derived from various mt genomes could be achieved by means of qRT PCR with specific primers and probes. Such experiments will contribute to a better understanding the consequences of heteroplasmy in plants.

## Conclusions

Our study of offspring from a single natural population of a gynodioecious species *S. vulgaris *revealed specific association of the transcription profiles of mt genes with mt DNA markers. The transcription profiles were not influenced by environment, but correlated with mt DNA haplotypes. We also did not detect any association between the gender and the transcription profile, which would suggest the role of a specific transcript in associated with differential CMS gene expression in the two genders. We found high between-family and limited within-family variation of mt DNA markers in the offspring under study. Within-sibship variation was attributable to paternal inheritance, lineage sorting or maybe to the rearrangement of mt DNA by recombination. Our results demonstrate that transcription of mt genes in *S. vulgaris *is very complex. Our studies indicate that naturally occurring mitochondrial rearrangements may have functional consequences in plants. It remains to be shown how variation in transcription may influence morphology or other fitness-related traits. An entire mt transcriptome should be considered to understand the role of mitochondria in determining a gender in this gynodioecious species.

## Methods

### Plant material

*Silene vulgaris *(Moench) Garcke (Caryophyllaceae) is a native Euro-Asiatic species that has been introduced to North America. In the summer of 2005, we collected ten maternal plants from the population Kováry Meadows (Czech Republic), located on the hillside 10 km west of Prague, at the altitude 300 m [17]. We sampled one branch carrying at least 15 mature capsules from each individual; individuals were separated by at least 10 m. The seeds were germinated in a greenhouse at the Institute of Experimental Botany AS CR, Prague, and the plants were grown under long days until flowering, when gender was determined. Flowers with at least two anthers were considered hermaphrodites, and those with less than two anthers were scored as females. A substantial portion of individuals changed gender in the course of cultivation. We have therefore distinguished the third gender category, shifting females. These were the hermaphrodite plants which produced female flowers at least once during the two years observation period. Most of the plants expressed flowers of only one gender at the same time, and a different gender appeared after stems were cut to near ground level and the plant regrew and flowered; however a few carried both female and hermaphrodite flowers at the same time.

### DNA amplification and sequencing

DNA was isolated from 1 g of fresh leaf or stem tissue using a sorbitol extraction method [29]. To identify restriction sites for PCR-RFLP markers, *cytochrome oxidase *1 (*cox1*) and *adenosine 5' triphosphate synthetase *subunit 1 (*atp1*) were PCR amplified from total DNA using primers published in [16] and [22], respectively. PCR primers were used to sequence the *cox1 *gene and internal primers were developed to sequence the *atp1 *gene. (AtpA297F: TCGACGTGTCGAAGTGAAAG; AtpA1170R: TCTGAGCCAAATTGAGCAAA). DNA nucleotide sequences of the *cox1 *and *atp1 *coding regions were determined for the maternal plants and a few representative progeny from each family. Sequences of mt genes were deposited in GenBank (EU805575 - EU805579). Based on sequence data, AluI and MspI restriction enzymes (Fermentas GmbH, Germany) were identified for discrimination among the *atp1 *PCR products, whereas MspI and DdeI were used to screen alleles in the *cox1 *PCR products [22].

### Heteroplasmy detection using "knock-back" approach

The "knock-back" approach developed by [22] was adopted to reveal additional rare copies of the *atp1 *gene in heteroplasmic individuals, which might have been overlooked by PCR-RFLP screening. The a *tp1 *haplotype KovA differs from haplotypes KovB and KovC by the presence of one SmaI site. When a plant is homoplasmic for the *atp1 *haplotype KovA, SmaI cuts all *atp1 *copies, revealing two bands on an agarose gel, and PCR amplification of genomic DNA pre-digested with SmaI does not generate any fragments. If the *atp1 *haplotypes KovB or KovC are found in very low copy number, however, SmaI restriction sites are not found in all copies of *atp1 *and PCR amplification of genomic DNA pre-digested by SmaI will be positive. For the knock-back analyses, we digested about 200 ng (1 μl) of genomic DNA with *SmaI *at 30°C in a final volume of 20 μl for 6 hours according to the manufacturer's directions (Fermentas). In a control reaction, water replaced the restriction enzyme. Two μl of digestion reaction mixture was used in PCR reactions with *atp1 *specific primers. The *atp1 *PCR fragment was produced if (1) genomic DNA contained rare copies of the *atp1 *gene different from the KovA haplotype, or if (2) genomic DNA of homoplasmic individual of the KovA haplotype was partially digested. As the case (2) represents an artifact and could be incorrectly interpreted as evidence for heteroplasmy; we confirmed the absence of SmaI site in *atp1 *PCR fragment by an additional SmaI digestion. Only those individuals, which provided an *atp1 *PCR fragment not cleavable by SmaI, were considered heteroplasmic.

### Southern hybridization

RFLP variation was assessed for the HindIII and EcoRI restriction sites flanking the *cox1 *and *atp1 *genes in two separate assays. This kind of mt markers is referred to as Southern-RFLP markers to distinguish them from PCR-RFLP markers located in the coding regions. Five hundred ng of genomic DNA was digested with either HindIII or EcoRI, electrophoretically separated overnight on a 0.9% agarose gel, transferred to a membrane and hybridized with non-radioactively labeled *cox1 *and *atp1 *probes as described by [17]. The completion of the digestion was checked by runnig an aliquot containing 50 ng of digested genomic DNA on a 0.9% agarose gel before the membrane transfer. Completely digested DNA was smeared. The membranes were usually stripped according to the manufacturer and rehybridized, so both *cox1 *and *atp1 *Southern-RFLP patterns were estimated using the same membrane. Relative intensity of individual bands in the same run was determined using Phosphoimager FLA7000.

Faint bands were present in many Southern-RFLP patterns, in varying positions. Because the post-hybridization washes were of very high stringency, these bands cannot represent non-specific targets. These bands identify *cox1 *or *atp1 *homologs, either full length or truncated, that are present either in the nucleus or in the mitochondrion, although the location in chloroplast cannot be also excluded. We also did not identify any *atp1 *or *cox1 *haplotypes that contained EcoRI or HindIII restriction sites, therefore one Southern band corresponds to one gene copy (full length or truncated).

### Northern hybridization

Total RNA was extracted from the flower buds (1 - 4 mm in size) of the greenhouse grown plants using an RNeasy Plant Mini kit (Qiagen). One μg of total RNA was loaded on agarose gel (1.8% agarose in 6.7% formaldehyde and 1 × MOPS buffer). RNA loading buffer was composed of 50% formamide (deionised), 6% formaldehyde, 1 × MOPS buffer, 10% glycerol and 0.05% bromophenolblue (w/v). After overnight electrophoresis, RNA was transferred to a positively charged membrane Hybond N+ (Amersham) by capillary blotting. The same probes as used for Southern hybridization were applied in EasyHyb buffer (Roche). Membranes were hybridized at 52°C overnight, washed at very high stringency (0.1 × SSC, 68°C), and detected using CDPStar (Roche) as a substrate. Exposure times of < 30 min were sufficient to detect strong bands on Hyperfilm (Amersham). If necessary, membranes were stripped with deionised formamide at 80°C according to the manufacturer's protocol.

## Authors' contributions

HOA performed Southern hybridizations and PCR-RFLP screens of *S. vulgaris *plants. KM ran and optimized Northern hybridizations and contributed to raw data interpretations. MSO helped to interpret the results and made large contributions to manuscript writing. HS collected the plant material, designed the experiments and drafted the manuscript. All authors read and approved the final manuscript.
